# Lard Injection Matrix for Serial Crystallography

**DOI:** 10.3390/ijms21175977

**Published:** 2020-08-19

**Authors:** Ki Hyun Nam

**Affiliations:** Department of Life Science, Pohang University of Science and Technology, Pohang 37673, Korea; structures@postech.ac.kr

**Keywords:** serial crystallography, serial millisecond crystallography, sample delivery, viscous medium, lard, fat

## Abstract

Serial crystallography (SX) using X-ray free electron laser or synchrotron X-ray allows for the determination of structures, at room temperature, with reduced radiation damage. Moreover, it allows for the study of structural dynamics of macromolecules using a time-resolved pump-probe, as well as mix-and-inject experiments. Delivering a crystal sample using a viscous medium decreases sample consumption by lowering the flow rate while being extruded from the injector or syringe as compared to a liquid jet injector. Since the environment of crystal samples varies, continuous development of the delivery medium is important for extended SX applications. Herein, I report the preparation and characterization of a lard-based sample delivery medium for SX. This material was obtained using heat treatment, and then the soluble impurities were removed through phase separation. The lard injection medium was highly stable and could be injected via a syringe needle extruded at room temperature with a flow rate < 200 nL/min. Serial millisecond crystallography experiments were performed using lard, and the room temperature structures of lysozyme and glucose isomerase embedded in lard at 1.75 and 1.80 Å, respectively, were determined. The lard medium showed X-ray background scattering similar or relatively lower than shortenings and lipidic cubic phase; therefore, it can be used as sample delivery medium in SX experiments.

## 1. Introduction

The serial crystallography (SX) technique allows for structure determination of a variety of materials, from macromolecules to small molecules, at room temperature [1,2,3]. Using an X-ray free electron laser (XFEL) with ultrashort pulse width or synchrotron X-rays for a short exposure duration, SX can significantly reduce radiation damage compared to conventional X-ray crystallography techniques [1,2,3]. In addition, the molecular dynamics of chemical or biological reactions of target samples can be observed through a time-resolved experiment using optical lasers or solutions [1,3]. Taken together, SX, using an XFEL or synchrotron X-ray, can be used to observe structural flexibility of molecules at room temperature and of radiation-sensitive materials (such as metalloproteins), in addition to the dynamics of molecules [3,4,5,6,7].

In the sample delivery process of typical SX, crystals are exposed only once to X-rays, or in the case of large crystals, a new volume is exposed to X-rays each time [8]. To collect a complete data set, numerous crystals are continuously delivered to the X-ray interaction point during data collection [8]. Various sample delivery methods, such as injectors [9,10], syringes [11,12,13], capillaries [14,15], fixed targets [16,17,18,19,20,21], and microfluidics [22,23], have been developed and applied to SX experiments; viscous materials can be used in combination with some sample delivery systems [15,24,25]. Crystal samples enclosed in viscous materials provide a stable injection stream even at low flow rates when extruded from an injector or syringe, thereby reducing sample consumption in the XFEL facility with low repetition rate or synchrotron [10,11,24,26,27,28,29,30,31,32,33]. In the capillary method with viscous medium, sample consumption can be reduced by allowing the sample to be delivered at a low flow rate by preventing the crystal sample from being sunk by gravity [15]. In addition, in a fixed-target sample holder for SX, a viscous material can hold the crystal samples of various shapes and sizes between two films [25].

In the SX experiment, viscous media used for sample delivery can be classified as amphiphilic, hydrophobic, or hydrophilic [24]. Amphiphilic sample delivery materials, such as lipidic cubic phase (LCP), can be used to deliver either membrane protein crystals grown in LCP or previously grown crystals of soluble proteins [10,34]. However, the phase can be changed depending on the solution content, temperature, and crystallization solution [35,36]. Meanwhile, LCP medium produces certain high background scattering [10]. Hydrophilic sample delivery materials, including agarose [26], hyaluronic acid [29], hydroxyethyl cellulose [30], sodium carboxymethyl cellulose [37], Pluronic F-127 [37], poly(ethylene oxide) [7], polyacrylamide [28], wheat starch [32], and alginate [32], have low background scattering compared to an LCP medium. Hydrophobic delivery materials, such as greases [11,29,30,33] and shortening [31], provide a stable injection stream due to viscous properties higher than other types of materials. Among them, shortening is a harmless material and therefore its injection matrix in SX is safe, easy to handle with temperature control, and has a lower cost than other delivery media [31]. However, although the melting temperature (Tm) of the shortening used in the experiment was higher than the room temperature, the stability of the shortening injection stream deteriorated because of heat generated from the beamline equipment during data collection in the experimental hutch [31]. Consequently, in this experiment, a stable shortening injection stream was produced when the temperature of the experimental hutch was maintained at 20 °C [31]. Although the SX application using shortening matrix was successfully proved, this material may be of limited use when the structure of the target material is to be observed above room temperature. Thus, it is worthwhile to develop materials with high Tm values, while maintaining the advantages of shortening injection streams, for use in SX.

Lard, a semi-solid white fatty substance obtained from pigs, is a stable substance used in food [38,39]. While this material includes all the previously reported advantages of shortening (safe, phase change with temperature control, and low cost), the melting point (36–42 °C) is higher [40], resulting in a stable injection stream at room temperature.

Herein, I report the preparation and characterization of lard as sample delivery media for SX experiments. These materials produce a stable injection stream at flow rates < 200 nL/min at room temperature. A serial millisecond crystallography (SMX) experiment was performed using lard, and the room temperature structure of lysozyme and glucose isomerase embedded in lard at 1.75 and 1.80 Å, respectively, was determined. The X-ray background scattering of lard was analyzed and compared with that of shortening and LCP. Lard can be applied as a sample delivery material in SX experiments.

## 2. Results

### 2.1. Preparation of Lard Material for SX Experiment

In a previous study, fat-based shortening materials required a data collection environment of approximately 20 °C to produce a stable injection stream at the experimental hutch [31]. To collect data at temperatures higher than room temperature while maintaining the advantages of shortening, lard materials with a higher Tm than shortening were selected. For the initial experiment, commercially available lard was purchased along with lard oil. However, this material exhibited a semi-solid state at a low temperature of 4 °C. Thus, it was difficult to use for experiments because it was close to the liquid state at room temperature.

**Figure 1:**
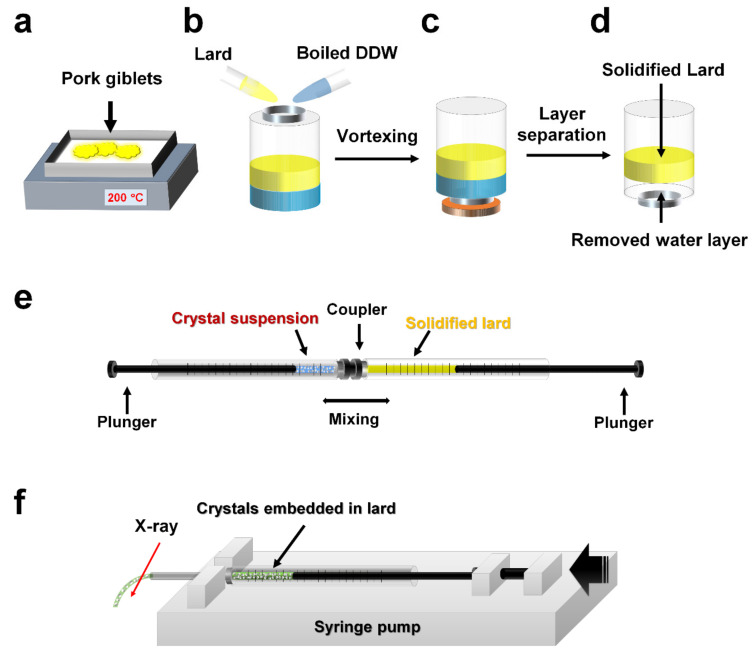
Schematic drawing of the preparation of lard injection matrix. (**a**) Lard extraction around pork giblets through heat treatment. (**b**) Mixing the lard extraction and boiled distilled deionized water (DDW). (**c**) Layer separation and solidification of lard by standing at room temperature. (**d**) Removing of the bottom water layer by opening the bottle cap. Boiled DDW was added again to repeat layer separation. (**e**) Mixing the crystals and lard in a dual syringe-setup. (**f**) Syringe containing the crystals embedded in lard was installed into syringe pump and delivered for serial millisecond crystallography.

Therefore, lard was isolated from pig fat tissue using a heat treatment which is typically used in food science [41,42]. Depending on the type of tissue, pig fat differs in composition and Tm value [43], and in this study, the fat around the intestines was used. Lard was separated from a pig’s intestines by heat treatment of 100 °C for 30 min (Figure 1a). These lard materials may have contained soluble substances [41], which could have caused physical damage to the crystal when mixed with the crystal sample. Accordingly, the separated fat was mixed with boiled distilled deionized water (DDW) and left to cool down at room temperature (Figure 1b). Then, fat and water formed layers because of density and polarity, and fat settled above the water, in which the soluble substances were dissolved (Figure 1c). When the temperature was cooled to room temperature, the upper layer of lard formed a solid, and the bottom water layer was removed (Figure 1d). The upper layer of lard part was mixed again with boiling DDW to perform layer separation five times to remove soluble impurities. The purified lard was dissolved by heating, transferred to a syringe, and then left to cool down at room temperature to solidify. The solidified lard was mixed with a crystal sample in a dual-syringe setup and then used in the experiment (Figure 1e,f). Since lard has a semi-solid nature at room temperature, it can be mixed with crystals like other viscous materials such as LCP or shortening.

### 2.2. Application of Lard Injection Matrix for SMX

**Figure 2:**
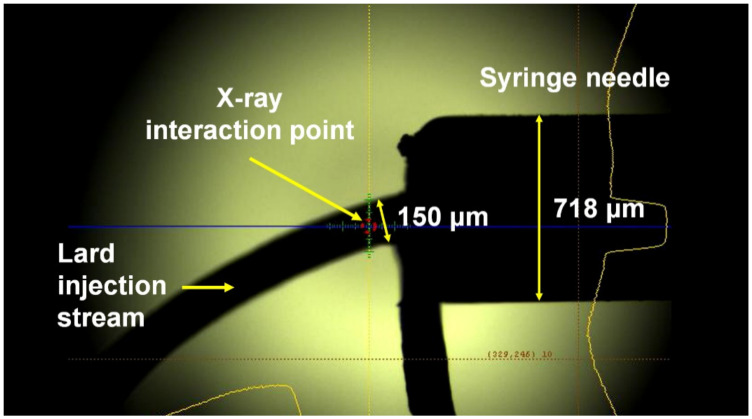
Snapshot of the lard injection stream embedding the lysozyme crystals. The 80% (*v*/*v*) lard delivery medium was extruded from a syringe needle with an inner diameter of 168 μm at room temperature.

To demonstrate whether SX studies can be performed at room temperature using lard, SMX data were collected at a synchrotron using lysozyme and glucose isomerase crystals as model samples. Crystals embedded in lard were delivered to the X-ray interaction point through a syringe needle with a 168 μm inner diameter (ID) (Figure 2 and Video S1). The diameter of the lard injection stream embedding the crystal samples was ~150 μm (Figure 2 and Video S1).

**Figure 3:**
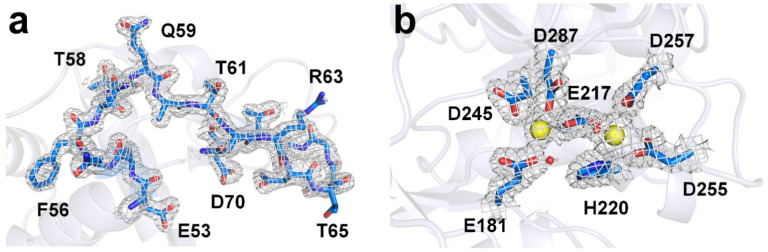
The 2Fo-Fc electron density map (grey mesh, 1.5 σ) of the room temperature structure of (**a**) lysozyme and (**b**) glucose isomerase delivered in lard. Blue sticks represent amino acids around active sites, while yellow spheres represent metal ions at the active site of glucose isomerase.

For lysozyme, a total of 60,000 images were collected, of which 16,136 images contained diffraction patterns. Among them, 14,365 images were indexed, and the indexing rate was 89.02%. Data were processed at 1.75 Å, and signal-to-noise ratio (SNR), R_split_, and Pearson’s correlation coefficients (CC) were 9.84, 7.21, and 0.9933, respectively (Table 1). The overall electron density map of lysozyme was very clearly observed (Figure 3a), and no significant radiation damage was observed around the disulfide-bonds (Cys24–Cys145, Cys48–Cys133, Cys82–Cys98, and Cys94–Cys112). The structure of lysozyme delivered to the lard showed high similarity to the structure of lysozyme at room temperature, delivered by polyacrylamide (PDB code 6IG6 and 6JXQ), shortenings (6KCB and 6KCD), agarose (6KD1 and 6LL3), wheat starch (7BVM), and alginate (7BVO), with an rms deviation of 0.092–0.221 Å for all Cα atoms.

For glucose isomerase, a total of 110,000 images were collected, of which 48,506 images contained the diffraction patterns. Among them, 18,889 images were indexed, and the indexing rate was 38.94%. Data were processed at 1.80 Å, and SNR, R_split_, and CC were 5.72, 12.90, and 0.9772, respectively (Table 1). The overall electron density map of glucose isomerase was very clearly observed (Figure 3b), and no significant radiation damage was observed around the metal binding active site. The structure of glucose isomerase delivered to lard showed high similarity to that of glucose isomerase at room temperature, delivered by shortenings (PDB code 6KCA and 6KCC), gelatin (6KD2 and 6LL2), wheat starch (7BVL), and alginate (7BVN), with an rms deviation of 0.082–0.385 Å for all Cα atoms.

### 2.3. Measurement of Background Scattering

During SMX data collection using lard material, unique background scattering from the lard delivery medium was observed. Although the crystal structure of lysozyme or glucose isomerase were successfully determined, the background scattering from lard can affect the signal-to-noise quality of diffraction data. Therefore, background scattering of lard was analyzed, because this analysis can be an important reference factor in the selection of delivery media.

**Figure 4:**
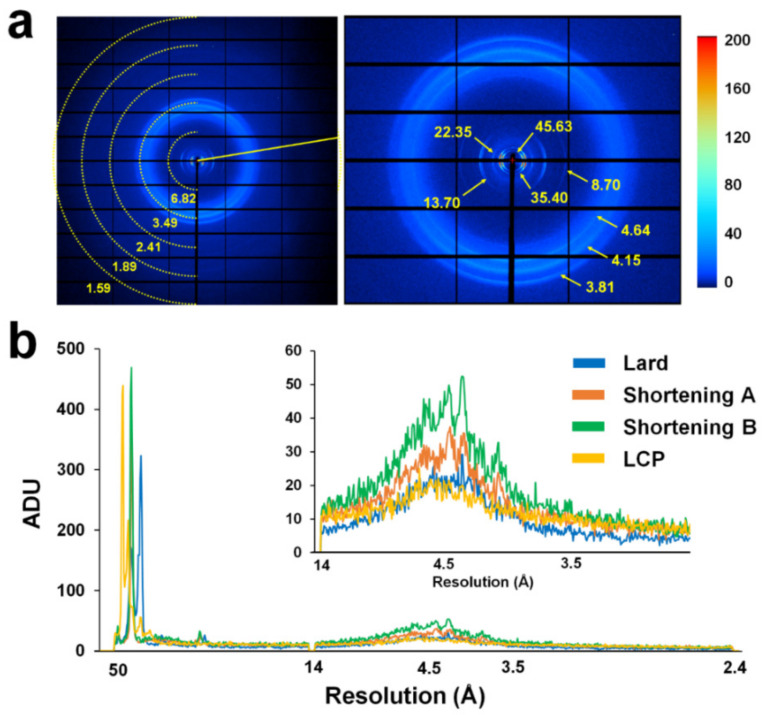
Measurement of X-ray background scattering of lard. (**a**) Typical scattering image of 80% (*w*/*v*) lard. (**b**) Two-dimensional profile of the average scattering intensities of 80% (*w*/*v*) lard, 80% (*w*/*v*) shortening A, 80% (*w*/*v*) shortening B, and lipidic cubic phases (LCP) (60% (*v*/*v*) monoolein).

Accordingly, X-ray background scattering for lard was comparatively analyzed with shortening A, shortening B, and LCP. Lard with a concentration of 80% (*v*/*v*), shortenings with 80% (*v*/*v*), and LCP (60% (*v*/*v*) monoolein) were delivered to the X-ray interaction point through a syringe needle with a 168 μm ID (Figure 4a), and the average intensities were analyzed from the beamstop to the 1.6 Å resolution (Figure 4b).

Background scattering from lard showed 172, 323, 24, 26, 16, and 15–30 analog-to-digital units (ADUs) at 45.63, 35.40, 22.35, 13.70, 8.70, and 3.5–5.0 Å areas, respectively (Figure 4a). The scattering of the ring patterns observed at 35.40, 22.35, and 8.70 Å formed a unique background scattering found only in lards, and the one at 3.5–3.8 Å was considered to include diffused scattering from the crystal solution along with lard-derived background scattering. Background scattering of the ring pattern observed in the lard was considered to be scattering by lipid packing in the lard. Background scattering of shortening A showed 371, 26.8, and 20–40 ADUs at 44, 14, and 3.8–4.5 Å areas, respectively. Background scattering of shortening B showed 469, 32.6, and 25–50 ADUs at 44, 14, and 3.8–4.5 Å areas, respectively. Background scattering of LCP showed 216 and 15–23 ADUs at 44 Å and 4.2–4.8 Å areas, respectively. Taken together, lard exhibited lower background scattering than shortenings at 14–3.0 Å and exhibited background scattering similar to that by LCP at 14.0–3.0 Å (Figure 4b).

## 3. Discussion

Shortening materials have been previously used as a stable injection stream in SMX experiments [31], but due to issues related to their Tm values, use at room temperature was limited. This study reported that lard can be used, at room temperature, as a delivery material for SX experiments while maintaining the advantages of shortening. Lard provided a stable injection stream at room temperature and could be successfully applied for room temperature structure determination. In this experiment, lard was extracted from three independent giblets to obtain lard extract. No significant differences were observed in injection stability and background scattering from the three samples. Meanwhile, if the direct extraction of lard is limited, the major constituents of lard, namely the palmitic, oleic, and linoleic acids, can be mixed and used in a similar manner to the lard composition ratio. Background scattering generated from lard did not significantly differ from shortenings or LCP, but it showed higher background scattering in a specific resolution region than hydrogel materials. Therefore, the use of lard may be limited to samples with weak diffraction intensity or in phasing experiments where SNR is important. Conversely, the crystal size and injection diameter used in this experiment were larger than those of a typical SX experiment. When delivering small crystals, it is important to provide a small inner diameter injection stream to minimize background scattering. On the other hand, the lard extracted in this experiment was probably not suitable for lipid-binding or lipolytic protein samples, since it contains several fats, such as oleic, palmitic, and linoleic acids, among others [44], and thus, may react with the crystal sample. Nevertheless, this study will not only expand the pool of viscous crystal delivery media for SX experiments but will also provide insights into the future of the development of fat-based delivery materials.

## 4. Materials and Methods

### 4.1. Preparation of Lard Injection Matrix

Fat around the pork giblets was obtained from a butcher shop. These fats were placed on a stainless-steel plate and heated to 100 °C to render fat. Next, the soluble impurities in the rendered lard were removed by phase separation method at room temperature using DDW. The extracted fat was put in a bottle with boiled DDW, vortexed, and left to cool down at room temperature (23–25 °C). When the lard solidified, the bottle cap was opened to remove the water layer. The solidified lard was heated to make a solution, and then the layer separation process was repeated five times by mixing with boiled DDW. Finally, the obtained lard was placed on a stainless-steel plate and heated to 100 °C to evaporate the residual moisture. Then, the lard solution was placed in a glass vial and stored at 4 °C.

### 4.2. Crystallization and Mixing of Crystals with the Lard Matrix

Sample preparation and crystallization methods of lysozyme and glucose isomerase have been reported previously [18,28]. Briefly, lysozyme, from egg white, was purchased from Hampton Research (HR7–110, Aliso Viejo, CA, USA) and dissolved in a buffer containing 10 mM Tris-HCl, pH 8.0, and 200 mM NaCl to make a final concentration of 50 mg/mL. Equal volumes (100 ul) of lysozyme solution and crystallization solution (100 μl) containing 0.1M Na-acetate, pH 4.6, 6% (*w*/*v*) PEG 8000, and 10% (*w*/*v*) NaCl were mixed in a 1.5 mL microcentrifuge tube, vortexed, and finally incubated overnight at 22 °C. Glucose isomerase, from *Streptomyces rubiginosus*, were purchased from Hampton Research (HR7-098, Aliso Viejo, CA, USA), which supplied the crystalline, and thus were directly used in the SX experiment without post-crystallization, as previously reported [31,32]. Products containing glucose isomerase crystals were stored at 4 °C for future use. The embedding process of the lysozyme and glucose isomerase crystals into the lard was conducted at room temperature. The crystal sizes of lysozyme and glucose isomerase were ~30 μm and <60 μm, respectively. The method of embedding crystals in the lard was the same as that used for the preparation of the previous shortening delivery method [31]. The solid lard contained in the glass vial was immersed in hot water (>100 °C) for 30 s, and the lard solution (40 μL) was transferred into a 100 μL syringe and left to cool down at room temperature until it solidified. The crystal suspension (30 µL) was transferred to another 100 µL syringe and left for 10 min in a vertical position, and then the supernatant was removed from the crystal suspension. The final 80% (*w*/*v*) lard delivery medium was used in the SMX experiment. The syringes containing the lard (40 µL) and crystal (10 µL) were connected using a syringe coupler, and then the suspension was gently mixed by moving it back and forth 30 times using a plunger. After moving the crystal embedded in lard to one syringe, the other syringe was removed and connected to a syringe needle with a 168 μm inner diameter (ID). This syringe crystal sample was installed in the syringe pump for sample delivery.

### 4.3. X-ray Data Collection

SMX experiments using a lard injection matrix were performed at the 11C beamline at Pohang Light Source II (Pohang, Korea) [45]. The X-ray beam size at the sample position was approximately 4 × 8 μm^2^ (vertical × horizontal: FWHM). The photon flux was 1.3 × 10^12^ photons/s, and the X-ray energy was 12.659 keV. Crystals embedded in lard were delivered by a syringe pump-based sample delivery method [13]. Samples were delivered at a flow rate of 200 nL/min using the Fusion Touch 100 syringe pump (CHEMYX, Stafford, TX, USA) through the syringe needle with an ID of 168 μm. Crystal samples embedded in lard medium were exposed to X-rays for 100 ms. Diffraction data were recorded on a Pilatus 6M with 10 Hz readout at 25–26 °C. For X-ray background scattering analysis of the viscous media, 20 images were randomly extracted from collected images, and the average intensity was measured in the range from the beamstop to 1.6 Å using the ADXV software (https://www.scripps.edu/tainer/arvai/adxv.html).

### 4.4. Structure Determination

Hit finding and image processing were performed using Cheetah [46] and CrystFEL [47] program, respectively. The electron density map was obtained by the molecular replacement method using phase-MR in Phenix [48]. The crystal structures of lysozyme (PDB code 6IG6) [28] and glucose isomerase (PDB code 5ZYC) [49] were used as the search models. The model building and refinement were accomplished using the COOT [50] and Phenix refinement in PHENIX [51], respectively. The geometry of final structures were validated using MolProbity [52]. Data collection and structure refinement statistics are shown in Table 1. Structure figures were prepared using the PYMOL program (https://pymol.org/). The structure factors and coordinates have been deposited in the Protein Data Bank under the accession code 7CJZ (lysozyme delivered in lard) and 7CK0 (glucose isomerase delivered in lard). Diffraction images and geometry files have been deposited in CXIDB under ID 153 (lysozyme derived in lard) and 154 (glucose isomerase delivered in lard).

## Figures and Tables

**Table 1:** Data collection and refinement statistics.

Data Collection	Lysozyme	Glucose Isomerase
Energy (eV)	12,659	12,659
Exposure time (ms)	100	100
Space group	P4_3_2_1_2	I222
Cell dimensions (Å)		
a	79.45	94.19
b	79.45	99.92
c	38.47	103.25
Collected images	60,000	110,000
Hits images	16,136	48,506
Indexed pattern	14,365	18,889
Resolution (Å)	80.0–1.75 (1.81–1.75)	72.46–1.80 (1.86–1.80)
Unique reflections	12,954 (1256)	45,422 (4492)
Completeness (%)	100.0 (100.0)	100.0 (100.0)
Redundancy	385.2 (266.8)	565.1 (387.8)
SNR	9.84 (3.60)	5.72 (2.81)
CC	0.9933 (0.8049)	0.9772 (0.5475)
CC*	0.9983 (0.9444)	0.9943 (0.8412)
R_split_ (%) ^a^	7.21 (33.84)	12.90 (39.09)
Wilson B factor (Å^2^)	33.85	30.62
**Refinement**		
Resolution (Å)	56.18–1.75	71.80–1.80
R_work_	16.62	15.81
R_free_^b^	18.65	17.66
Rms deviations		
Bond length (Å)	0.014	0.007
Bond angle (°)	1.937	0.975
B factors (Å^2^)		
Protein	39.29	29.95
Ligands	42.42	22.70
Water	41.71	39.43
Ramachandran (%)		
Preferred	98.43	96.61
Allowed	1.57	3.12
Outliers	0.00	0.26

Values for the outer shell are given in parentheses. ^a^*R_split_* = 12·∑hklIhkleven−Ihklodd12Ihkleven−Ihklodd. ^b^ R_free_ was calculated as R_work_ using a randomly selected subset (10%) of unique reflections not used for structure refinement.

## Supplementary Materials

The following are available online at https://www.mdpi.com/1422-0067/21/17/5977/s1.
